# Comparative Evaluation of a Locally Formulated Subclinical Mastitis Test Reagent Against the California Mastitis Test (CMT) in Dairy Cows in Ethiopia

**DOI:** 10.1002/vms3.70499

**Published:** 2025-07-10

**Authors:** Sisay Weldegebriel Zeweld, Enquebaher Kassaye Tarekegn

**Affiliations:** ^1^ Department of Veterinary Public Health and Food Safety College of Veterinary Sciences, Mekelle University Mekelle Ethiopia

**Keywords:** California mastitis test, dairy cows, diagnostic accuracy, Ethiopia, Ethiopian mastitis test, subclinical mastitis

## Abstract

Subclinical mastitis (SCM) remains a major productivity‐limiting disease in dairy cattle, particularly in low‐resource settings where diagnostic access is limited. The California mastitis test (CMT) is widely used for on‐farm SCM detection but is increasingly inaccessible in Ethiopia due to high costs and supply constraints. This study aimed to evaluate the diagnostic performance of a newly formulated, low‐cost Ethiopian mastitis test (EMT) reagent as an alternative to CMT under field conditions. A cross‐sectional study was conducted from October 2024 to February 2025 in Mekelle, Ethiopia, involving 112 lactating dairy cows across diverse farm settings. Milk samples were tested using both EMT and CMT reagents, and somatic cell count (SCC) served as the reference standard. Diagnostic agreement was assessed using sensitivity, specificity, predictive values, Kappa statistics and receiver‐operating characteristic (ROC) analysis. EMT demonstrated a sensitivity of 90.14%, specificity of 100%, positive predictive value of 100% and negative predictive value of 85.42%. The Kappa value (*κ* = 0.87) indicated strong agreement with CMT. ROC analysis confirmed high diagnostic accuracy (area under the curve [AUC] = 0.908), although slightly lower than CMT (AUC = 1.0). EMT also showed strong associations with established SCM risk factors, such as cow age, breed, hygiene, nutrition and housing. These findings validate EMT as a reliable, field‐adaptable and economically feasible diagnostic tool for SCM. Its adoption could enhance udder health surveillance, reduce reliance on imports and promote sustainable dairy production in Ethiopia. National scale‐up and broader ecological validation are recommended to support policy integration and widespread implementation.

## Graphical abstract

A novel Ethiopian mastitis test (EMT), made from local ingredients, was evaluated against the gold‐standard CMT for detecting subclinical mastitis in dairy cows. EMT showed 90.14% sensitivity, 100% specificity and strong agreement (*κ* = 0.87) with CMT. It offers a cost‐effective, accessible and field‐adaptable solution. EMT enhances smallholder dairy productivity, curbs antimicrobial misuse and supports local veterinary innovation. This reliable, scalable test presents a transformative tool for mastitis control in the resource‐limited settings of Ethiopia.

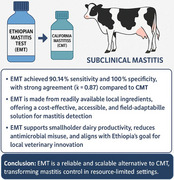

## 1 Introduction

### 1.1 Background and Justification

Mastitis remains one of the most economically devastating diseases affecting dairy cattle worldwide, characterized by inflammation of the mammary gland, primarily due to bacterial infection (Haider et al. 2023; Narváez‐Semanate et al. 2022; Dego 2020). It manifests in two forms: clinical mastitis, which presents with observable signs such as swelling, redness and abnormal milk and subclinical mastitis (SCM), which lacks visible symptoms but is associated with elevated somatic cell counts (SCCs) in milk (Tripathi et al. 2017). SCM is particularly problematic due to its silent progression and high prevalence, often going undetected while causing significant reductions in milk yield and quality, increased intramammary transmission risk and eventual economic losses to dairy producers.

Globally, early and accurate detection of SCM is crucial for minimizing these impacts. The California mastitis test (CMT) is one of the most widely adopted on‐farm diagnostic tools, offering a rapid and practical estimation of SCC through the observation of gel formation after reagent interaction with milk (Roberts 2025; Rochmah et al. 2024; Saidani and Zeroual 2024). However, despite its proven utility, the routine use of CMT in low‐resource settings like Ethiopia remains limited. The high cost of the imported reagent, exceeding 6000 Ethiopian birr per litre, along with inconsistent market availability, poses significant barriers to its widespread application (Moje and Abebaw 2024; Rust et al. 2023; Balemi et al. 2021; Birhanu et al. 2017). In recent years, CMT availability in Ethiopia has declined further due to supply chain interruptions and foreign currency constraints, making the search for locally produced alternatives more urgent.

SCM is not only prevalent in Ethiopia but also across sub‐Saharan Africa, where it affects 60%–80% of lactating cows depending on management practices (Mgonja et al. 2023; Khasapane et al. 2023; Ndahetuye et al. 2021). Yet, the region continues to face a shortage of affordable, rapid diagnostic tools that can be used reliably under field conditions. In Ethiopia, livestock production is integral to rural livelihoods, food security and the national economy. Dairy farming plays a particularly important role, serving as a major source of nutrition and income for millions of households (Abebe et al. 2023; Girma and Tamir 2022; Zeryehun and Abera 2017; Mekonnen et al. 2017). Nevertheless, diseases like SCM undermine the productivity of the dairy sector. Studies report that SCM can lead to milk yield losses as high as 70%, increased veterinary costs, milk discards and premature culling of animals (Birhanu et al. 2017; Ibrahim et al. 2023; Demil et al. 2022; Mulshet et al. 2017; Geleta et al. 2019). The lack of affordable and accessible diagnostic tools further exacerbates these challenges, particularly among smallholder farmers, who are the backbone of the Ethiopian dairy industry (Muturi 2020; Getaneh and Gebremedhin 2017; Hamadani et al. 2013).

To address these challenges, alternative approaches have been explored. Studies investigating the use of local detergents as CMT substitutes reported varying diagnostic performances. Ethiopian detergents demonstrated sensitivities ranging from 28% to 75% and high specificities between 84% and 98%, indicating some potential for local application (Rust et al. 2023). However, Nigerian detergents performed better, showing sensitivities of 68%–80% and specificities of 93%–97%, comparable to commercial CMT reagents. Further innovations, such as the use of *Hibiscus sabdariffa* extract or simplified field tests like the surf field mastitis test (SFMT), have also shown promising results in rural settings (Muhammad et al. 2010; Leach et al. 2008). Despite these efforts, challenges remain in standardizing these alternatives, as test performance often varies with detergent composition and operator interpretation. Moreover, none of these substitutes have been formally validated or registered at the national level in Ethiopia, limiting their regulatory uptake and consistent use.

Another critical aspect of accurate SCM diagnosis is its role in antimicrobial stewardship. Inaccurate or unavailable testing often leads to the blind use of antibiotics, which contributes to the growing global concern of antimicrobial resistance (AMR) in both veterinary and human health settings (Paramasivam et al. 2023; Kovačević et al. 2022; Abed et al. 2021). Thus, reliable field‐based mastitis tests can help farmers and veterinarians make evidence‐based treatment decisions, reducing the misuse of antibiotics and preserving their efficacy.

Recognizing these limitations, a novel diagnostic reagent, the Ethiopian mastitis test (EMT), was developed to provide a locally formulated, cost‐effective and field‐adaptable alternative to the commercial CMT. The EMT reagent is composed of double‐distilled water, detergent‐based lytic and emulsifying agents (sodium laureth sulphate, ethoxylated alcohol, dimethyl aminoethyl methacrylate and sodium chloride) and a food‐grade colourant for enhanced visual interpretation. Developed by Dr. Sisay Weldegebriel Zeweld and registered with the Ethiopian Intellectual Property Office (ET/UM/2015/1802), EMT aims to offer a reliable, affordable and scalable solution for SCM detection in Ethiopian dairy farms. Its formulation enables visual clarity, ease of use and rapid application even by minimally trained users, making it especially suitable for low‐literacy or rural farm settings.

Beyond its technical merits, the EMT also contributes to broader goals of import substitution of Ethiopia, local innovation and livestock development. As the country pursues greater self‐reliance in animal health tools, EMT offers a sustainable, homegrown solution that can support smallholder productivity and national food security. Thus, this study was designed to scientifically evaluate the diagnostic performance of the EMT reagent in comparison to the standard CMT, contributing to the development of sustainable, locally available mastitis control strategies.

### 1.2 Objective

The specific objective of this study was to evaluate the sensitivity, specificity and diagnostic agreement of a locally formulated SCM test reagent compared to the CMT.

## 2 Materials and Methods

### 2.1 Study Area

The study was conducted in Mekelle city, the capital of the Tigray region in Northern Ethiopia. Mekelle is situated at an altitude of approximately 2200 m above sea level, with a semi‐arid climate characterized by moderate rainfall (averaging 500–600 mm annually) and average temperatures ranging from 12°C to 28°C. The city is a key hub for dairy production in the region, with a growing number of both smallholder and semi‐commercial dairy farms contributing to the local milk supply. Dairy farming in Mekelle is typically characterized by intensive and semi‐intensive production systems, utilizing both local and crossbred cows. Farmers often face challenges related to animal health management, feed availability and limited access to diagnostic tools, making the area an ideal setting for evaluating low‐cost and field‐adaptable solutions like the locally formulated SCM test reagent (Berhe et al. 2024). The subcities selected for the study, Ayder, Hawelti, Adi Haqi, Hadnet, Kedamay‐weyane, Quiha and Semien, represent a diversity of farm sizes, management systems and geographic distribution within the city.

### 2.2 Study Design

A cross‐sectional study design was employed to assess the diagnostic performance of a locally formulated SCM test reagent in comparison to the CMT. The study was conducted from October 2024 to February 2025 across selected dairy farms in Mekelle city, situated in the Tigray region of Northern Ethiopia.

### 2.3 Study Population

The study population included lactating dairy cows of various breeds and ages managed under different production systems.

### 2.4 Sampling and Sample Size Determination

The required sample size was calculated using the formula described by Thrusfield (2018) for estimating prevalence in a finite population. A previous prevalence of SCM in Mekelle city (54.4%) was used as the expected prevalence by Gebrekrustos et al. (2012), with a 95% confidence interval and 5% desired absolute precision. First, the initial sample size without considering the finite population was calculated using the following formula:

$$n=\frac{Z^{2}\cdot P\left(1-P\right)}{d^{2}}$$

where *Z* = 1.96 (for 95% CI), *P* = 0.544 and *d* = 0.05. Substituting these values gave 380.8. Because the total number of milking cows in the study population was *N* = 158, the finite population correction was applied to obtain the required sample size (*n*_r_) using the following formula:

$$n_{r}=\frac{1}{1/n-1/N}$$

After substituting the values, the final adjusted sample size was 112 milking cows, which were randomly selected from the total population across the identified dairy farms across seven subcities in Mekelle. Specifically, 18 cows were sampled from Ayder (8 farms, 25 milking cows), 14 from Hawelti (7 farms, 20 cows), 19 from Adi‐Haqi (9 farms, 27 cows), 21 from Hadnet (10 farms, 30 cows), 12 from Kedamay‐weyane (6 farms, 17 cows), 13 from Quiha (6 farms, 18 cows) and 15 from Semien (8 farms, 21 cows). In total, the study covered 158 milking cows in the subcity populations.

### 2.5 Study Methods

#### 2.5.1 Sample Collection Procedure

Milk samples were aseptically collected from all four quarters of each selected cow. The teats were first cleaned and dried, and the foremilk was stripped before sampling to minimize contamination, and 1 mL of milk was collected into sterile CMT paddles for immediate testing and gently mixed with an equal volume of the test reagent (Roberts 2024; Rathish and Chandran 2024). Each sample was labelled with a unique cow ID, quarter number, date and farm location.

#### 2.5.2 Diagnostic Testing

**FIGURE 1:**
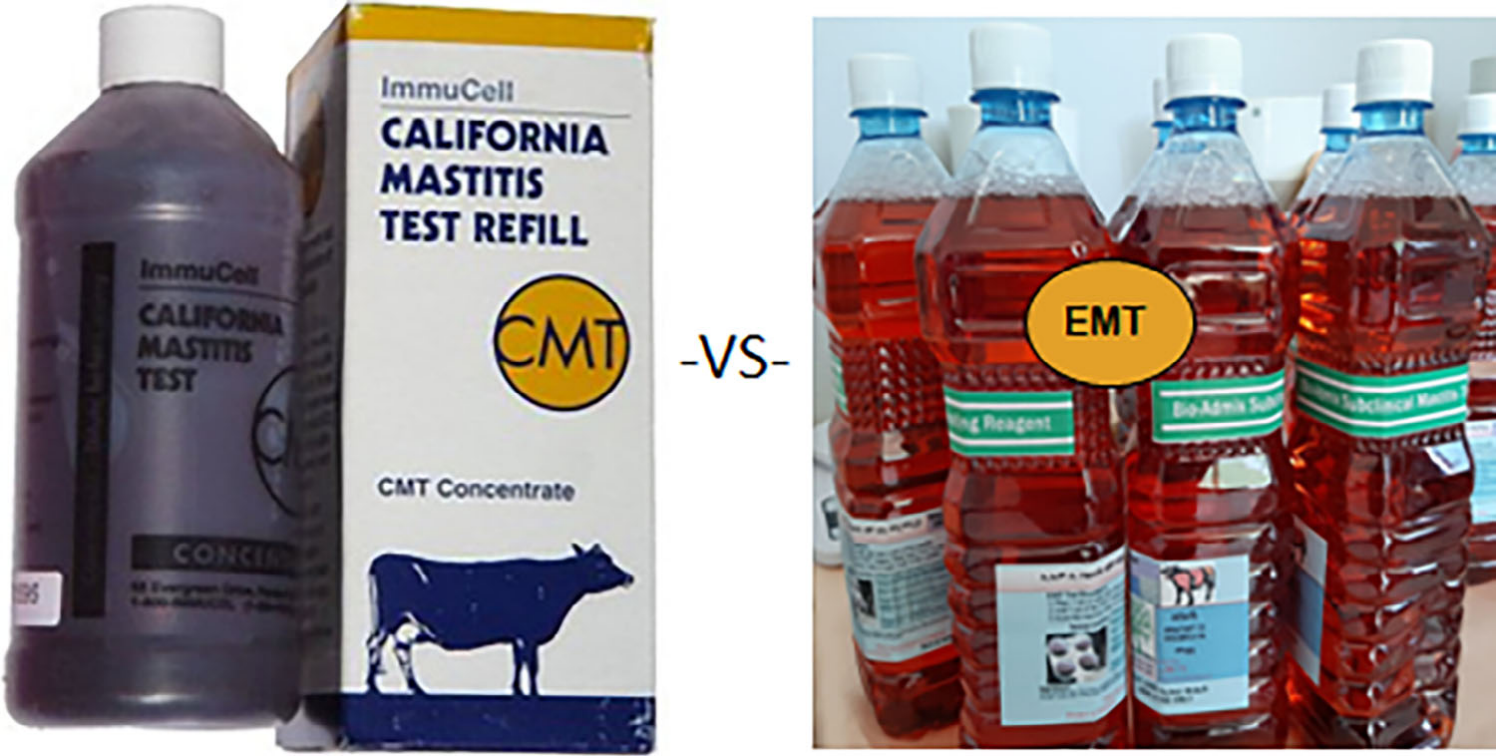
Demonstration of CMT and EMT reagents used in the study. CMT, California mastitis test; EMT, Ethiopian mastitis test.

CMT was used as the reference test. Equal volumes of milk and a commercially available CMT reagent (Neogen/GEA Farm Technologies, Product No. 4999‐1180‐026, USA) were mixed in a paddle (Roberts 2024, 2025). The degree of gel formation was visually scored on a scale of 0–3 (0 = negative, 1 = trace, 2 = moderate, 3 = strong positive) according to standard interpretation guidelines (Adkins and Middleton 2017; Ruegg 2017; Schukken et al. 2003; Gamroth and Adams 1981) (Table 1). Using the same procedure and milk samples, the locally developed reagent called EMT reagent was tested under identical conditions (Figure 1). Equal parts of milk and the local reagent were mixed and scored using the same visual grading scale to ensure consistency in data comparison.

**TABLE 1:** Interpretation of somatic cell count (SCC) levels for detection of subclinical mastitis (SCM).

Test result	SCC range (cells/mL)	Interpretation
Negative	<200,000	Healthy udder, no infection
Trace	200,000–400,000	Possible early infection or stress
Moderately Positive (+)	400,000–1,200,000	Likely infection, mild mastitis
Strongly Positive (++, +++)	>1,200,000	Definite infection, severe mastitis

*Source*: Adkins and Middleton (2017), Ruegg (2017), Schukken et al. (2003) and Gamroth and Adams (1981).

#### 2.5.3 Validation via SCC

To ensure the accuracy and reliability of the locally formulated SCM test reagent, a subset of milk samples was selected for validation based on divergent results observed between the locally formulated test reagent (EMT) and the CMT. These samples were subjected to SCC analysis, which serves as the gold standard for diagnosing SCM (Fonseca et al. 2025; Hisira et al. 2023; Niasari‐Naslaji et al. 2016). SCC is a critical indicator of inflammation in the udder and is widely used to assess the presence and severity of mastitis, with higher cell counts correlating with more severe infections. The SCC was measured using microscopic methods. By comparing the results from the EMT and the CMT with SCC values, we were able to assess the sensitivity, specificity and diagnostic agreement of the test reagent relative to the established SCC threshold for SCM.

To determine the SCC via the direct microscopic method, 0.05 mL milk sample was first taken from each cow (Sun et al. 2023). The sample was mixed with a staining solution, Methylene blue, to stain the somatic cells, which consist mainly of white blood cells and epithelial cells (Jose et al. 2022). A milk smear was prepared by placing a drop of the stained milk sample on a microscope slide, spreading it evenly and allowing it to air‐dry. After drying, the smear was examined under a microscope at 400× magnification. The somatic cells were counted manually in multiple fields of view to ensure accuracy, and the total count was then converted into cells per millilitre (cells/mL) using a standard calculation. This method served as a reference to validate the test results for SCM, allowing comparison with the CMT results (Jose et al. 2022; Ferronatto et al. 2018).

#### 2.5.4 Questionnaire Survey

A structured questionnaire was administered to dairy farm owners or attendants to collect data on a range of categorical variables potentially associated with the occurrence of SCM. The survey captured the subcity location of each farm (Ayder, Hawelti, Adi Haqi, Hadnet, Kedamay‐weyane, Quiha and Semien) and farm size, classified as small‐scale (<10 cows), medium‐scale (10–30 cows) or large‐scale (>30 cows). Milking frequency (once, twice or thrice daily) was recorded, and respondents were also asked about teat hygiene practices, categorized as poor (no or irregular cleaning), fair (cleaning with water only) or good (cleaning with disinfectant after each milking). The presence or absence of teat injuries was also noted. Cow‐level data collected included age categories (young: <3 years, adult: 3–7 years, older: >7 years), breed type (local, crossbreed, exotic) and parity (primiparous, biparous or multiparous). Information was gathered on lactation stage (early: 1–3 months, mid: 4–6 months, late: >6 months or dry period) and milk yield levels (low: <10 L/day, medium: 10–20 L/day, or high: >20 L/day). The questionnaire also assessed housing conditions (poor, fair or good), nutritional management practices (poor, moderate or good) and cow stress levels (high, moderate or low), including potential sources of stress such as overcrowding and handling. Data on the use of antibiotics (yes or no) and observed udder health status (healthy, mastitis suspected or confirmed mastitis) were collected.

## 3 Statistical Analysis

Data collected from both the EMT reagent and the commercial CMT reagent were analysed to assess their diagnostic performance, with SCC (cell/mL) as the dependent variable and various categorical variables from the questionnaire survey as independent variables. Descriptive statistics, including frequencies and percentages, were first used to summarize the distribution of the test results from both reagents using SPSS Version 20. A 2 × 2 contingency table was constructed to evaluate the diagnostic agreement between the EMT reagent and the CMT reagent (Table 2).

**TABLE 2:** Evaluation of diagnostic agreement between Ethiopian mastitis test (EMT) and California mastitis test (CMT) reagents using a 2 × 2 contingency table.

	CMT‐positive	CMT‐negative	Total
EMT‐positive	*a*	*b*	*a + b*
EMT‐negative	*c*	*d*	*c + d*
Total	***a + c***	***b + d***	***a + b + c + d***

Diagnostic performance indicators were calculated on the basis of the contingency table to evaluate the locally formulated EMT reagent. Sensitivity (true positive rate) was calculated as *a*/(*a + c*), measuring the ability of the EMT to correctly identify mastitis‐positive samples. Specificity (true negative rate) was computed as *d*/(*b* + *d*), assessing the ability of the reagent to correctly detect mastitis‐negative samples. The positive predictive value (PPV), defined as *a*/(*a* + *b*), indicated the probability that a sample testing positive truly had mastitis. Conversely, the negative predictive value (NPV), calculated as *d*/(*c* + *d*), represented the probability that a sample testing negative was truly free of infection.

The Kappa statistic (*κ*) was employed to quantify the level of agreement beyond chance between the EMT and the reference CMT. Interpretation of *κ* values followed the guidelines described by Munoz and Bangdiwala (1997), where *κ* = 0 indicated no agreement, *κ* = 0.2–0.3 indicated moderate agreement, *κ* = 0.5–0.6 indicated substantial agreement and *κ* = 0.8 or greater indicated almost perfect agreement. This evaluation was critical for assessing the consistency between the EMT and CMT in detecting SCM. Receiver‐operating characteristic (ROC) curve analysis was performed to further assess the diagnostic accuracy of both tests. The area under the ROC curve (area under the curve [AUC]) was calculated as a summary measure of each test's overall performance, with AUC values approaching 1.0 indicating excellent discriminatory ability and values near 0.5 suggesting no discriminatory power (Zhou et al. 2014). Additionally, Chi‐square (*χ* ^2^) tests were conducted to evaluate associations between categorical variables obtained from the questionnaire survey and the presence of SCM as determined by both the EMT and CMT. This analysis facilitated the identification of significant risk factors associated with elevated SCCs and SCM status. A *p* value of <0.05 was considered statistically significant, with 95% confidence intervals used for all estimates.

## 4 Results

### 4.1 Descriptive Statistics and Associations With SCM

The association between various factors and the prevalence of SCM, as assessed by both the CMT and the EMT, was evaluated using chi‐squared (*χ* ^2^) tests. Significant associations were observed for several factors. Age had a highly significant relationship with mastitis, with older cows (≥7 years) exhibiting 100% positivity for both CMT (*χ* ^2^ = 27.606, *p* < 0.001) and EMT (*χ* ^2^ = 27.61, *p* < 0.001). Breed also showed a strong association with mastitis, where crossbred and exotic breeds (Zebu X Holstein‐Friesian) were more likely to test positive compared to indigenous breeds (*χ* ^2^ = 20.02, *p* < 0.001 for both CMT and EMT). Furthermore, cows subjected to poor teat hygiene (*χ* ^2^ = 52.88, *p* < 0.001) and those with teat injuries (*χ* ^2^ = 31.91, *p* < 0.001) had significantly higher mastitis prevalence for both tests (Table 3).

**TABLE 3:** Association of categorical variables with subclinical mastitis (SCM) as assessed by California mastitis test (CMT) and Ethiopian mastitis test (EMT).

Variable	Category	CMT‐positive (%)	EMT‐positive (%)	*χ* ^2^ (df)	*p* value (CMT)	*p* value (EMT)
Age	Young (<3 years)	21 (77.8%)	21 (77.8%)	27.61 (2)	<0.001	<0.001
	Adult (3–7 years)	26 (42.6%)	19 (31.1%)			
	Old (>7 years)	24 (100%)	24 (100%)			
Breed of milking cow	Local breeds (indigenous cattle or Zebu)	0	0	20.02 (2)	<0.001	<0.001
	Crossbreeds (Zebu X HF)	47 (42.0%)	40 (38.4%)			
	Exotic breeds (Holstein‐Friesian or HF)	24 (21.4%)	24 (21.4%)			
Milking frequency	Twice a day	69 (61.6%)	62 (55.4%)	8.21 (2)	0.016	0.032
	Thrice a day	2 (1.8%)	2 (1.8%)			
	Once a day	0	0			
Teat hygiene	Poor hygiene (no cleaning, or irregular cleaning)	37 (33.0%)	37 (33%)	52.88 (2)	<0.001	<0.001
	Fair hygiene (cleaning with water only)	34 (30.4%)	27 (24.1%)			
	Good hygiene (cleaning with disinfectant)	0	0			
Presence of teat injury	Yes (presence of teat injuries)	37 (33%)	37 (33%)	31.91 (1)	<0.001	<0.001
	No (absence of teat injuries)	34 (30.4%)	27 (24.1%)			
Udder health status	Healthy	13 (11.6%)	6 (5.4%)	69.47 (1)	<0.001	<0.001
	Suspected mastitis	58 (51.8%)	58 (51.8%)			
Housing conditions	Poor (overcrowded, damp, insufficient bedding)	46 (41.1%)	45 (40.2%)	60.38 (1)	<0.001	<0.001
	Fair (adequate space, but minor issues	25 (22.6%)	18 (16.1%)			
	Good (spacious, dry, clean and well‐ventilated)	0	0			
Nutritional management	Poor (lack of balanced diet, inadequate supplementation)	47 (42.0%)	48 (42.9%)	50.01 (1)	<0.001	<0.001
	Moderate (adequate, but not optimal diet)	22 (19.6%	16 (14.3%			
	Good (well‐balanced diet, nutritional supplements)	2 (1.8%)	1 (0.9%)			
Stress	High stress (transport, poor handling, overcrowding)	24 (21.4%)	24 (21.4%)	24.16 (1)	<0.001	<0.001
	Moderate stress (minor handling issues)	47 (42%)	40 (35.7%)			
	Low stress (calm, handled well)	0	0			
Antibiotic use	Yes (improper use)	71 (63.4%)	64 (62.5%)	37.14 (1)	<0.001	<0.001
	No	0	0			
Parity	Primiparous	22 (19.6%)	21 (18.8%)	15.35 (1)	<0.001	<0.001
	Biparous	25 (22.3%)	19 (17%)			
	Multiparous	24 (21.4%)	24 (21.4%)			
Lactation stage	Early lactation (1–3 months)	24 (21.4%)	24 (21.4%)	4.51 (1)	0.034	0.035
	Mid lactation (4–6 months)	0	0			
	Late lactation (7+ months)	47 (42%)	40 (35.7%)			
Milk yield	Low milk yield (<10 L)	7 (6.2%)	0	5.87 (1)	0.015	0.015
	Moderate milk yield (10–20 L)	30 (26.8%)	30 (26.8%)			
	High Milk Yield (>20 L)	34 (30.4%)	34 (30.4%)			

Other environmental and management‐related factors also demonstrated significant associations. Housing conditions, particularly poor housing, were strongly correlated with mastitis, with cows in substandard housing showing higher positivity rates (*χ* ^2^ = 60.38, *p* < 0.001). Similarly, stress levels and nutritional management played crucial roles in mastitis prevalence, with cows experiencing moderate to high stress (*χ* ^2^ = 24.16, *p* < 0.001) and those receiving poor nutrition (*χ* ^2^ = 50.01, *p* < 0.001) exhibiting higher rates of infection. Additionally, antibiotic use was significantly associated with mastitis, where frequent or improper use was linked to increased positivity rates (*χ* ^2^ = 37.14, *p* < 0.001). The milking frequency, lactation stage and milk yield were also significantly related to mastitis occurrence. Cows milked twice a day had higher mastitis prevalence (*χ* ^2^ = 8.21, *p* = 0.016 for CMT; *p* = 0.032 for EMT), whereas cows in early lactation were more likely to test positive (*χ* ^2^ = 4.512, *p* = 0.034 for CMT; *p* = 0.035 for EMT). Moreover, cows with low milk yield (<10 L) had a significantly higher likelihood of mastitis (*χ* ^2^ = 5.87, *p* = 0.015 for both tests). These findings underline the multifaceted nature of SCM and the importance of both management and biological factors in its prevalence (Table 3).

### 4.2 Comparison of CMT and EMT for Diagnosing SCM in Dairy Cows

The performance of the CMT and the EMT in detecting SCM was evaluated using 112 milk samples collected from lactating cows. No missing data were recorded. On the basis of the CMT, 63.4% (71/112) of samples tested positive for SCM, whereas 36.6% (41/112) tested negative. Similarly, the EMT identified 57.1% (64/112) of samples as positive and 42.9% (48/112) as negative. Cross‐tabulation analysis revealed that all samples negative by CMT (41/41; 100%) were also negative by EMT, demonstrating complete agreement for negative results. Among samples testing positive by CMT, 90.1% (64/71) were confirmed positive by EMT, whereas 9.9% (7/71) were negative. Statistical analysis showed a highly significant association between the two tests, as evidenced by the Pearson Chi‐square test (*χ* ^2^ = 86.235, df = 1, *p* < 0.001) and Fisher's exact test (*p* < 0.001). These findings indicate a strong concordance between the CMT and EMT in diagnosing SCM. Although the CMT exhibited a slightly higher detection rate than the EMT, the strong agreement between the two tests suggests that the EMT could serve as a reliable and practical alternative to the CMT under field conditions (Table 4).

**TABLE 4:** Cross‐tabulation and statistical association between California mastitis test (CMT) and Ethiopian mastitis test (EMT) results.

CMT result	EMT‐negative (*n*, %)	EMT‐positive (*n*, %)	Total (*n*, %)	*χ* ^2^	Fisher's exact test	df	*p* value
Negative	41 (36.6)	0 (0.0)	41 (36.6)	86.24	82.60	1	< 0.001
Positive	7 (6.2)	64 (57.1)	71 (63.4)				
**Total**	**48 (42.9)**	**64 (57.1)**	**112 (100.0)**				

### 4.3 Diagnostic Performance and Agreement of CMT and EMT

The diagnostic performance of the EMT was assessed in comparison to the CMT using a 2 × 2 contingency table, and key statistical measures of agreement were calculated. In the analysis, 64 samples that tested positive for SCM by both EMT and CMT (*a*) were identified, whereas there were no false positives (*b* = 0). Of the 41 samples that tested negative by CMT, all were also negative by EMT (*d*), whereas 7 samples tested negative by CMT but positive by EMT (*c*). The total number of samples was 112 (Table 5).

**TABLE 5:** Diagnostic agreement between Ethiopian mastitis test (EMT) and California mastitis test (CMT) for subclinical mastitis (SCM) detection.

	CMT‐positive	CMT‐negative	Total
EMT‐positive	*a* = 64	*b* = 0	*a* + *b* = 64
EMT‐negative	*c* = 7	*d* = 41	*c* + *d* = 48
**Total**	***a* + *c* = 71**	***b* + *d* = 41**	**112**

The sensitivity of the EMT was calculated as the proportion of true positives correctly identified by the test. This value is given by the following formula: sensitivity = *a*/(*a* + *c*), yielding a sensitivity of 90.14%. This indicates that 90.14% of the true SCM cases were identified by EMT. The specificity of the EMT refers to the proportion of true negatives correctly identified by the test. It is calculated as specificity = *d*/(*b* + *d*), resulting in a specificity of 100%. This shows that all the samples that were negative for mastitis by CMT were also correctly identified as negative by the EMT.

The PPV indicates the likelihood that a positive test result accurately reflects the presence of disease. It is given by PPV = *a*/(*a* + *b*), which results in a PPV of 100%. This means that all positive EMT results were true positives for SCM when compared to the CMT. The NPV represents the likelihood that a negative test result is truly negative and is calculated as NPV = *d*/(*c* + *d*). The NPV for the EMT was found to be 85.42%, meaning that 85.42% of the samples testing negative for SCM by EMT were also negative by CMT. Finally, the Kappa statistic (*κ*) was calculated to assess the agreement between the two tests beyond what would be expected by chance. The formula for the Kappa statistic is *κ* = (observed agreement − expected agreement)/(1 − expected agreement). The observed agreement is calculated as (*a* + *d*)/total samples, and the expected agreement is determined on the basis of the proportions of each category in the contingency table. The Kappa value of 0.87 indicates a high level of agreement between the EMT and CMT, suggesting that the two tests are in strong concordance, beyond what would be expected by chance. These results demonstrate that the EMT performs well in detecting SCM, with high sensitivity, specificity, PPV and NPV, and exhibits a substantial level of agreement with the CMT, making it a reliable alternative for mastitis detection in field conditions (Table 5).

### 4.4 SCC Validation Results

Descriptive statistical analysis was conducted on the SCC (cells/mL) data obtained from 112 samples. The SCC exhibited a wide range of 255,675 cells/mL, with values varying from a minimum of 54,325 cells/mL to a maximum of 310,000 cells/mL. The mean SCC across the samples was 185,179.28 cells/mL, indicating the average level of somatic cells present in the milk samples analysed. The standard deviation was 102,005.649 cells/mL, suggesting a substantial spread of the data around the mean. In this study, the diagnostic performance of SCC was evaluated using two reagents, the CMT and the EMT, for the detection of SCM in dairy cows.

**FIGURE 2:**
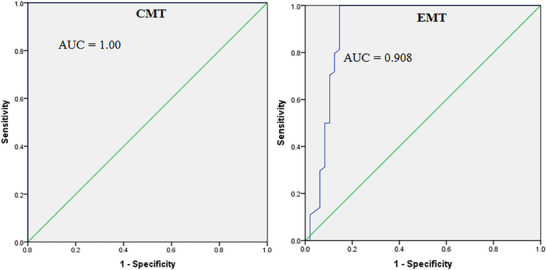
ROC curve analysis of CMT and EMT using SCC. AUC, area under the curve; CMT, California mastitis test; EMT, Ethiopian mastitis test.

ROC curve analysis was conducted for both tests using SPSS Version 20. For the CMT, the AUC was 1.00, indicating a perfect diagnostic accuracy with complete separation between positive and negative cases. The sensitivity and specificity were both 100% at a cutoff value of 128,762.5 cells/mL and remained high across several thresholds, suggesting that SCC when interpreted through CMT is an excellent marker for SCM without any overlap between diseased and healthy groups. In contrast, the EMT demonstrated an AUC of 0.908, reflecting a very good but slightly lower diagnostic performance compared to the CMT. Although the EMT exhibited high sensitivity at lower cutoff values, a drop in specificity was observed, with some overlap between positive and negative cases. The existence of ties between positive and negative groups suggests potential variability or limitations in the precision of the EMT reagent. Despite this, the EMT still provides strong diagnostic utility, achieving over 90% overall accuracy but with slightly less perfect discrimination compared to the CMT. Overall, these findings suggest that while both tests are useful for detecting SCM based on SCC, the CMT offers superior diagnostic performance, achieving perfect classification, whereas the EMT shows very good but slightly less precise diagnostic accuracy (Figure 2).

## 5 Discussion

This study represents the first scientific evaluation of a locally formulated SCM screening reagent, the EMT, as an alternative to the widely used CMT in Ethiopia. The EMT was developed in response to the urgent need for affordable, accessible and field‐adaptable diagnostic tools, particularly for smallholder dairy farmers who are disproportionately affected by the high prevalence of SCM and the prohibitive cost and unavailability of imported CMT reagents. The innovation of EMT not only fills a critical gap in the veterinary diagnostic landscape of the country but also aligns with national efforts to promote local solutions for livestock health management.

The EMT demonstrated excellent diagnostic performance, with a sensitivity of 90.14%, specificity of 100% and a Kappa value of 0.87, indicating strong agreement with the CMT. The PPV (100%) and NPV (85.42%) further confirm the reliability of EMT in detecting SCM cases under field conditions. These results are consistent with the ROC analysis, which showed an AUC of 0.908, suggesting excellent discriminatory power and confirming the robustness of EMT in distinguishing between mastitis‐positive and mastitis‐negative samples. Although the CMT achieved a perfect AUC of 1.00, its availability and affordability are increasingly problematic in Ethiopia due to import constraints, limited distribution channels and prices exceeding 6000 Ethiopian birr per litre (Moje and Abebaw 2024; Rust et al. 2023).

Previous efforts to identify alternative diagnostic reagents in Ethiopia, particularly the use of local detergents, have shown inconsistent results. For example, Rust et al. (2023) reported a wide sensitivity range (28%–75%) and specificities between 84% and 98% depending on the detergent brand and operator interpretation. Although some Nigerian studies found better results using household detergents (sensitivity 68%–80%, specificity 93%–97%) (Muhammad et al. 2010), none approached the diagnostic agreement or consistency observed with EMT in this study. Other experimental field‐level alternatives, such as *H. sabdariffa* extract or the SFMT, have also shown potential (Leach et al. 2008) but lack formulation standardization and commercial feasibility.

In this study, EMT also reflected strong associations with known mastitis risk factors, such as older age, crossbreeding, poor teat hygiene, teat injuries, poor housing, low nutrition and high stress levels, mirroring patterns observed in previous studies (Ibrahim et al. 2023; Demil et al. 2022; Mulshet et al. 2017; Geleta et al. 2019). This alignment not only validates the reliability of EMT in epidemiological assessments but also underscores its potential as a practical surveillance tool at the farm level.

Although EMT missed 7 of the 71 mastitis‐positive cases (false negatives), this limitation may be attributed to subjective visual scoring, subtle differences in somatic cell thresholds, or milk sample variation. The EMT, like all visually interpreted gel‐based tests, can be influenced by environmental lighting, sample temperature or operator skill (Adkins and Middleton 2017; Ruegg 2017). Despite this, the complete absence of false positives and perfect specificity provide a strong advantage over both unstandardized local detergents and even some commercial alternatives.

In practical terms, EMT offers a highly feasible and scalable solution to a longstanding diagnostic barrier in Ethiopia. Its ingredients are locally accessible, the reagent is easy to prepare and interpret and the cost is dramatically lower than imported CMT kits. The visual clarity of the reagent, ensured by the inclusion of food‐grade colourants, further supports its use by farmers or veterinary health workers with minimal training.

Moreover, in the current context where CMT reagents are scarce, expensive and largely unavailable, the EMT stands out as the best available alternative. It is not merely a substitute but a strategically superior solution that supports the goals of Ethiopia for veterinary self‐sufficiency, livestock productivity and rural economic empowerment. This is particularly important for smallholder dairy systems, where mastitis control is essential to prevent economic losses, milk wastage and unnecessary antibiotic usage (Abebe et al. 2023; Girma and Tamir 2022; Muturi 2020). However, a few limitations must be acknowledged. The study was conducted in a single urban area (Mekelle), and future research should extend to diverse agro‐ecological zones to confirm generalizability. Furthermore, quantitative evaluation tools, such as digital scoring systems or automated somatic cell counters, could enhance the precision and objectivity of the EMT. Long‐term shelf‐life studies, reagent stability tests and user feedback assessments should also be explored in future phases.

## 6 Conclusion and Recommendations

This study provides robust evidence supporting the diagnostic validity and operational feasibility of the EMT as a reliable alternative to the CMT for the detection of SCM in dairy cows under Ethiopian field conditions. The EMT demonstrated a high sensitivity (90.14%), perfect specificity (100%) and excellent diagnostic agreement with the CMT (*κ* = 0.87). ROC analysis further confirmed the strong diagnostic performance of EMT, with an AUC of 0.908. These results underscore the ability of EMT reagent to accurately differentiate between mastitis‐positive and mastitis‐negative milk samples, with diagnostic characteristics approaching those of the gold‐standard CMT. Importantly, EMT achieved this performance using a locally formulated, low‐cost reagent composed of readily available ingredients, thereby addressing the persistent challenges of affordability and accessibility associated with imported CMT reagents in Ethiopia.

Beyond its diagnostic accuracy, the EMT aligns with Ethiopia's strategic goals of fostering local innovation, reducing dependence on foreign imports and enhancing veterinary public health infrastructure. The test is easy to use, requires minimal training and provides visually interpretable results, making it particularly suitable for use by smallholder farmers, community animal health workers and field veterinarians in resource‐limited settings. The complete specificity of the test and its ability to detect mastitis patterns consistent with known risk factors (e.g., age, breed, hygiene, housing and nutrition) further validate its epidemiological reliability and practical relevance.

On the basis of these findings, several recommendations are warranted. First, national and regional livestock health authorities should prioritize the formal endorsement and wide‐scale adoption of the EMT as a frontline diagnostic tool for SCM detection in Ethiopia. This includes its integration into routine mastitis surveillance programmes and on‐farm herd health monitoring strategies. Second, efforts should be made to institutionalize the local production and distribution of EMT reagent through public–private partnerships to ensure quality control, scalability and sustained availability. Third, targeted training programmes should be implemented to build the capacity of end‐users, including farmers, animal health extension workers and veterinary professionals, to standardize test application and interpretation procedures. Lastly, future studies should validate EMT performance across different agro‐ecological zones and dairy production systems and assess its shelf‐life stability and usability under various environmental conditions. The EMT represents a scientifically validated, context‐appropriate and economically viable solution for SCM diagnosis. Its adoption can play a transformative role in improving udder health, enhancing milk quality, promoting rational antimicrobial use and ultimately boosting the productivity and sustainability of the dairy sector of Ethiopia.

## Author Contributions

**Sisay Weldegebriel Zeweld**: conceptualization, methodology, investigation, data curation, formal analysis, writing – original draft, visualization and project administration. **Enquebaher Kassaye Tarekegn**: supervision, validation, writing – review and editing.

## Ethics Statement

Due to the recent conflict, the Animal Ethical Committee at Mekelle University College of Veterinary Sciences was not fully established at the time of the study. Hence, formal ethical clearance was not obtained. However, all procedures followed standard veterinary ethical practices, and verbal consent was obtained from farm owners who voluntarily participated. A request for retrospective clearance has been submitted.

## Conflicts of Interest

The authors declare no conflicts of interest.

## Peer Review

The peer review history for this article is available at https://publons.com/publon/10.1002/vms3.70499.

## Data Availability

All data generated or analysed during this study are included in this published article. Additional datasets are available from the corresponding author on reasonable request.

## References

[vms370499-bib-0001] Abebe, R. , A. Markos , M. Abera , and B. Mekbib . 2023. “Incidence Rate, Risk Factors, and Bacterial Causes of Clinical Mastitis on Dairy Farms in Hawassa City, Southern Ethiopia.” Scientific Reports 13, no. 1: 10945. 37414815 10.1038/s41598-023-37328-1 PMC10326075

[vms370499-bib-0002] Abed, A. H. , A. M. Menshawy , M. M. Zeinhom , et al. 2021. “Subclinical Mastitis in Selected Bovine Dairy Herds in North Upper Egypt: Assessment of Prevalence, Causative Bacterial Pathogens, Antimicrobial Resistance and Virulence‐Associated Genes.” Microorganisms 9, no. 6: 1175. 34072543 10.3390/microorganisms9061175 PMC8229104

[vms370499-bib-0003] Adkins, P. R. , and J. R. Middleton . 2017. Laboratory Handbook on Bovine Mastitis. National Mastitis Council, Incorporated.

[vms370499-bib-0004] Balemi, A. , B. Gumi , K. Amenu , et al. 2021. “Prevalence of Mastitis and Antibiotic Resistance of Bacterial Isolates From CMT Positive Milk Samples Obtained From Dairy Cows, Camels, and Goats in Two Pastoral Districts in Southern Ethiopia.” Animals 11, no. 6: 1530. 34073967 10.3390/ani11061530 PMC8225129

[vms370499-bib-0005] Berhe, A. , M. Balehegn , S. Abera , D. Kiros , and G. Beyene . 2024. “Assessment of GHG Emission From Dairy Cattle Manure Management Practices in Rural and Urban Dairy Production in Enderta District and Mekelle City, Northern Ethiopia.” East African Journal of Veterinary and Animal Sciences 8, no. 1: 1–10.

[vms370499-bib-0006] Birhanu, M. , S. Leta , G. Mamo , and S. Tesfaye . 2017. “Prevalence of Bovine Subclinical Mastitis and Isolation of Its Major Causes in Bishoftu Town, Ethiopia.” BMC Research Notes 10: 1–6. 29268785 10.1186/s13104-017-3100-0 PMC5740909

[vms370499-bib-0007] Dego, O. K. 2020. Bovine Mastitis: Part I. Animal Reproduction in Veterinary Medicine. IntechOpen.

[vms370499-bib-0008] Demil, E. , L. Teshome , Y. Kerie , et al. 2022. “Prevalence of Subclinical Mastitis, Associated Risk Factors and Antimicrobial Susceptibility of the Pathogens Isolated From Milk Samples of Dairy Cows in Northwest Ethiopia.” Preventive Veterinary Medicine 205: 105680. 35691136 10.1016/j.prevetmed.2022.105680

[vms370499-bib-0009] Ferronatto, J. A. , T. C. Ferronatto , M. Schneider , et al. 2018. “Diagnosing Mastitis in Early Lactation: Use of Somaticell, California Mastitis Test and Somatic Cell Count.” Italian Journal of Animal Science 17, no. 3: 723–729.

[vms370499-bib-0010] Fonseca, M. , D. Kurban , J. P. Roy , et al. 2025. “Usefulness of Differential Somatic Cell Count for Udder Health Monitoring: Identifying Referential Values for Differential Somatic Cell Count in Healthy Quarters and Quarters With Subclinical Mastitis.” Journal of Dairy Science 108, no. 4: 3917–3928. 39694240 10.3168/jds.2024-25403

[vms370499-bib-0011] Gamroth, M. J. , and H. P. Adams . 1981. The California mastitis test . Oregon State University.

[vms370499-bib-0012] Gebrekrustos, M. , B. Afera , and H. Tassew . 2012. “Prevalence of Mastitis and Its Relationship With Risk Factors in Smallholder Dairy Farms in and Around Mekelle.” Red Veterinaria Electronica 13, no. 9: 46.

[vms370499-bib-0013] Geleta, B. , D. Beyene , A. Wubete , and F. Abunna . 2019. “Sub Clinical Mastitis in Dairy Farms of Addis Ababa and Sebeta Towns, Ethiopia.” Biological and Medical Journal of Science and Technology Research 12, no. 5: 9566–9571.

[vms370499-bib-0014] Getaneh, A. M. , and E. Z. Gebremedhin . 2017. “Meta‐Analysis of the Prevalence of Mastitis and Associated Risk Factors in Dairy Cattle in Ethiopia.” Tropical Animal Health and Production 49: 697–705. 28185209 10.1007/s11250-017-1246-3

[vms370499-bib-0015] Girma, A. , and D. Tamir . 2022. “Prevalence of Bovine Mastitis and Its Associated Risk Factors Among Dairy Cows in Ethiopia During 2005–2022: A Systematic Review and Meta‐Analysis.” Veterinary Medicine International 2022, no. 1: 7775197. 36164492 10.1155/2022/7775197 PMC9509276

[vms370499-bib-0016] Haider, A. , M. Ikram , I. Shahzadi , and M. Asif Raza . 2023. “Bovine Mastitis.” In Polymeric Nanoparticles for Bovine Mastitis Treatment, 49–80. Springer.

[vms370499-bib-0017] Hamadani, H. , A. Khan , M. Banday , et al. 2013. “Bovine Mastitis‐A Disease of Serious Concern for Dairy Farmers.” International Journal of Livestock Research 3, no. 1: 42–55.

[vms370499-bib-0018] Hisira, V. , F. Zigo , M. Kadaši , et al. 2023. “Comparative Analysis of Methods for Somatic Cell Counting in Cow's Milk and Relationship Between Somatic Cell Count and Occurrence of Intramammary Bacteria.” Veterinary Sciences 10, no. 7: 468. 37505872 10.3390/vetsci10070468 PMC10384197

[vms370499-bib-0019] Ibrahim, N. , F. Regassa , T. Yilma , and T. Tolosa . 2023. “Impact of Subclinical Mastitis on Uterine Health, Reproductive Performances and Hormonal Profile of Zebu× Friesian Crossbred Dairy Cows in and Around Jimma Town Dairy Farms, Ethiopia.” Heliyon 9, no. 6: e16793. 37303553 10.1016/j.heliyon.2023.e16793 PMC10250799

[vms370499-bib-0020] Jose, K. R. , K. Vijayakumar , K. J. Davis , and V. Shyma . 2022. “Evaluation of Two Different Methods to Estimate the Somatic Cell Count of Bovine Milk.” JIVA 20, no. 1: 39.

[vms370499-bib-0021] Khasapane, N. G. , C. Byaruhanga , O. Thekisoe , S. J. Nkhebenyane , and Z. T. Khumalo . 2023. “Prevalence of Subclinical Mastitis, Its Associated Bacterial Isolates and Risk Factors Among Cattle in Africa: A Systematic Review and Meta‐Analysis.” BMC Veterinary Research 19, no. 1: 123. 37573335 10.1186/s12917-023-03673-6 PMC10422699

[vms370499-bib-0022] Kovačević, Z. , M. Samardžija , O. Horvat , et al. 2022. “Is There a Relationship Between Antimicrobial Use and Antibiotic Resistance of the Most Common Mastitis Pathogens in Dairy Cows?” Antibiotics 12, no. 1: 3. 36671204 10.3390/antibiotics12010003 PMC9854474

[vms370499-bib-0023] Leach, K. , M. Green , J. Breen , et al. 2008. “Use of Domestic Detergents in the California Mastitis Test for High Somatic Cell Counts in Milk.” Veterinary Record 163, no. 19: 566–570. 18997186 10.1136/vr.163.19.566

[vms370499-bib-0024] Mekonnen, S. A. , G. Koop , S. T. Melkie , C. D. Getahun , H. Hogeveen , and T. J. Lam . 2017. “Prevalence of Subclinical Mastitis and Associated Risk Factors at Cow and Herd Level in Dairy Farms in North‐West Ethiopia.” Preventive Veterinary Medicine 145: 23–31. 28903872 10.1016/j.prevetmed.2017.06.009

[vms370499-bib-0025] Mgonja, F. R. , M. M. Charles , and A. S. Katakweba . 2023. “Prevalence of Subclinical Mastitis and Associated Risk Factors in Dairy Cattle From Institution Farms in Morogoro Municipality.” World Journal of Veterinary Science 4, no. 1: 1020.

[vms370499-bib-0026] Moje, N. , and B. Abebaw . 2024. “Bovine Mastitis: *Staphylococcus aureus* Isolation and Identification From Small Holder Dairy Farms Located in and Around Hawassa Town, Southern Ethiopia.” East African Journal of Biophysical and Computational Sciences 5, no. 1: 40–50.

[vms370499-bib-0027] Muhammad, G. , A. Naureen , M. N. Asi , and M. Saqib . 2010. “Evaluation of a 3% Surf Solution (Surf Field Mastitis Test) for the Diagnosis of Subclinical Bovine and Bubaline Mastitis.” Tropical Animal Health and Production 42: 457–464. 19731065 10.1007/s11250-009-9443-3

[vms370499-bib-0028] Mulshet, Y. , S. Derso , and A. Nigus . 2017. “Prevalence of Bovine Subclinical Mastitis and Associated Risk Factors in Addis Ababa, Central Ethiopia.” Online Journal of Animal and Feed Research 7, no. 5: 124–133.

[vms370499-bib-0029] Munoz, S. R. , and S. I. Bangdiwala . 1997. “Interpretation of Kappa and B Statistics Measures of Agreement.” Journal of Applied Statistics 24, no. 1: 105–112.

[vms370499-bib-0030] Muturi, E. 2020. “Effect of Mastitis on Milk Production in Dairy Cows in Kenya.” Journal of Animal Health 2, no. 1: 85–91.

[vms370499-bib-0031] Narváez‐Semanate, J. L. , C. A. Daza‐Bolaños , C. E. Valencia‐Hoyos , D. T. Hurtado‐Garzón , and D. C. Acosta‐Jurado . 2022. “Diagnostic Methods of Subclinical Mastitis in Bovine Milk: An Overview.” Revista Facultad Nacional De Agronomía Medellín 75, no. 3: 10077–10088.

[vms370499-bib-0032] Ndahetuye, J. B. , M. Leijon , R. Båge , K. Artursson , and Y. Persson . 2021. “Genetic Characterization of *Staphylococcus aureus* From Subclinical Mastitis Cases in Dairy Cows in Rwanda.” Frontiers in Veterinary Science 8: 751229. 34869725 10.3389/fvets.2021.751229 PMC8637448

[vms370499-bib-0033] Niasari‐Naslaji, A. , H. Pezeshk , A. Atakpour , et al. 2016. “Estimation of Somatic Cell Count, as Gold Standard to Detect Subclinical Mastitis, in Dromedary Camel.” Journal of Camel Practice and Research 23, no. 1: 175–178.

[vms370499-bib-0034] Paramasivam, R. , D. R. Gopal , R. Dhandapani , et al. 2023. “Is AMR in Dairy Products a Threat to Human Health? An Updated Review on the Origin, Prevention, Treatment, and Economic Impacts of Subclinical Mastitis.” Infection and Drug Resistance 16: 155–178. 36636377 10.2147/IDR.S384776 PMC9831082

[vms370499-bib-0035] Rathish, R. , and A. Chandran . 2024. “Detection of Mastitis Milk.” In Analytical Methods for Milk and Milk Products, 21–38. Apple Academic Press.

[vms370499-bib-0036] Roberts, J. 2024. “CPD Article: The California Mastitis Test: What Is the Value?” Livestock 29, no. 5: 184–193.

[vms370499-bib-0037] Roberts, J. 2025. “The California Mastitis Test: What Is the Value?” Livestock 30, no. S2: S4–S10.

[vms370499-bib-0038] Rochmah, E. R. , D. Raharjo , S. Hidanah , M. H. Effendi , A. M. Witaningrum , and S. H. Warsito . 2024. “Effectiveness of the California Mastitis Test (CMT), Reductase Test, and Alcohol Test for Dairy Cows Subclinical Mastitis Detection.” Jurnal Agro Veteriner (Agrovet) 7, no. 2: 91–97.

[vms370499-bib-0039] Ruegg, P. L. 2017. “A 100‐Year Review: Mastitis Detection, Management, and Prevention.” Journal of Dairy Science 100, no. 12: 10381–10397. 29153171 10.3168/jds.2017-13023

[vms370499-bib-0040] Rust, J. D. , M. J. Christian , C. J. Vance , et al. 2023. “A Study of the Effectiveness of a Detergent‐Based California Mastitis Test (CMT), Using Ethiopian and Nigerian Domestic Detergents, for the Detection of High Somatic Cell Counts in Milk and Their Reliability Compared to the Commercial UK CMT.” Gates Open Research 5: 146. 37362381 10.12688/gatesopenres.13369.2 PMC10285044

[vms370499-bib-0041] Saidani, K. , and F. Zeroual . 2024. “Detection of Bovine Mastitis Using the California Mastitis Test Under Field Conditions in Algeria.” Revue D'élevage Et De Médecine Vétérinaire Des Pays Tropicaux 77: 37426.

[vms370499-bib-0042] Schukken, Y. , D. Wilson , F. Welcome , L. Garrison‐Tikofsky , and R. Gonzalez . 2003. “Monitoring Udder Health and Milk Quality Using Somatic Cell Counts.” Veterinary Research 34, no. 5: 579–596. 14556696 10.1051/vetres:2003028

[vms370499-bib-0043] Sun, X. , R. Zhao , N. Wang , et al. 2023. “Milk Somatic Cell Count: From Conventional Microscope Method to New Biosensor‐Based Method.” Trends in Food Science & Technology 135: 102–114.

[vms370499-bib-0044] Thrusfield, M. 2018. Veterinary Epidemiology. John Wiley & Sons.

[vms370499-bib-0045] Tripathi, S. , N. Arora , S. Shekhar , and V. Rajora . 2017. “Prevalence, Bacterial Association and In Vitro Antimicrobials Susceptibility of Subclinical Mastitis in Crossbred Cows.” International Journal of Current Microbiology and Applied Sciences 6, no. 10: 2722–2726.

[vms370499-bib-0046] Zeryehun, T. , and G. Abera . 2017. “Prevalence and Bacterial Isolates of Mastitis in Dairy Farms in Selected Districts of Eastern Harrarghe Zone, Eastern Ethiopia.” Journal of Veterinary Medicine 2017, no. 1: 6498618. 28352648 10.1155/2017/6498618 PMC5352971

[vms370499-bib-0047] Zhou, X. H. , N. A. Obuchowski , and D. K. McClish . 2014. Statistical Methods in Diagnostic Medicine. John Wiley & Sons.

